# Supplementary material for: Splice-Junction-Based Mapping of Alternative Isoforms in the Human Proteome
Source: Cell Rep. Author manuscript; Available in PMC 2020 Jan 15. (PMC6961840; doi:10.1016/j.celrep.2019.11.026)
Supplement: 3 [file NIHMS1546469-supplement-3.zip › DF2/PXD0010154/Pancreas-82-Q5VZR4-AGAQAAGSTWVLHETFSQH.pdf]

A

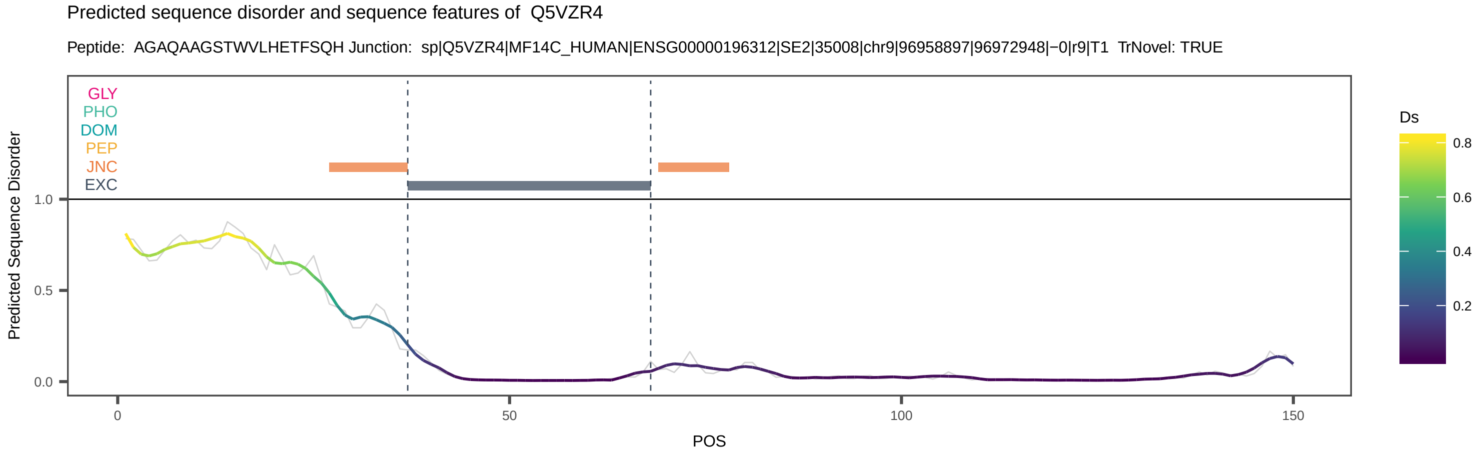
Predicted sequence disorder and sequence features of Q5VZR4
Peptide: AGAQAAGSTWVLHETFSQH Junction: sp|Q5VZR4|MF14C_HUMAN|ENSG00000196312|SE2|35008|chr9|96958897|96972948|−0|r9|T1 TrNovel: TRUE
GLY
PHO
DOM
PEP
JNC
EXC
Predicted Sequence Disorder
1.0
0.5
0.0
0
50
100
150
POS
Ds
0.8
0.6
0.4
0.2

B

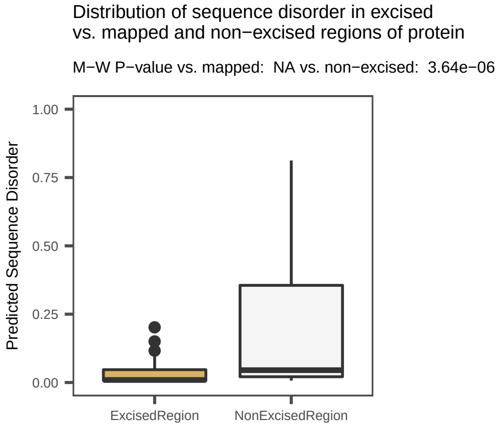
Distribution of sequence disorder in excised vs. mapped and non−excised regions of protein
M−W P−value vs. mapped: NA vs. non−excised: 3.64e−06
Predicted Sequence Disorder
1.00
0.75
0.50
0.25
0.00
ExcisedRegion
NonExcisedRegion

C
